# Assessing Community Vulnerability over 3 Waves of COVID-19 Pandemic, Hong Kong, China

**DOI:** 10.3201/eid2707.204076

**Published:** 2021-07

**Authors:** Qiuyan Liao, Meihong Dong, Jiehu Yuan, Richard Fielding, Benjamin J. Cowling, Irene Oi Ling Wong, Wendy Wing Tak Lam

**Affiliations:** University of Hong Kong, Hong Kong, China (Q. Liao, M. Dong, J. Yuan, R. Fielding, B.J. Cowling, I.O.L. Wong, W.W.T. Lam);; Hong Kong Science and Technology Park, Hong Kong (B.J. Cowling)

**Keywords:** COVID-19, SARS-CoV-2, coronavirus, 2019 novel coronavirus disease, severe acute respiratory syndrome coronavirus 2, zoonoses, coronavirus disease, viruses, vaccine-preventable diseases, coronavirus infection, pandemics, community vulnerability, pandemic impact, Hong Kong, China

## Abstract

We constructed a coronavirus disease community vulnerability index using micro district-level socioeconomic and demographic data and analyzed its correlations with case counts across the 3 pandemic waves in Hong Kong, China. We found that districts with greater vulnerability reported more cases in the third wave when widespread community outbreaks occurred.

The coronavirus disease (COVID-19) pandemic disproportionally affects socially disadvantaged populations because of economic, social, and demographic factors, as well as their health conditions and practices (*1*). Identifying vulnerable communities and effectively allocating ameliorating resources to them are necessary if policy makers are to manage the effects of COVID-19. Community vulnerability indexes (CVIs) have been increasingly used to assess community social vulnerability to a pandemic using community-level socioeconomic and demographic data (*2*–*7*). In the United States, greater CVI and vulnerability in domains of minority status, household composition, housing, transportation, and disability at the county level were significantly associated with greater risk of COVID-19 diagnosis (*3*,*4*). We aimed to construct a CVI more socioculturally adapted to metropolises in Asia to explain the impact of COVID-19 across more microgeographic units (i.e., districts) within a highly urbanized city, Hong Kong, China. We also analyzed the extent that CVI was correlated with the evolution of the COVID-19 pandemic in Hong Kong.

## The Study

Hong Kong has long been regarded as an epicenter for many infectious diseases and is predisposed to severe COVID-19 impact because of its dense and rapidly aging population (*8*,*9*). Geographically, Hong Kong comprises 3 main regions, New Territories, Kowloon, and Hong Kong Island, which are further subdivided into 18 administrative districts (*10*). As of August 31, 2020, Hong Kong had experienced 3 waves of COVID-19 (Appendix Figure 1), reporting 4,811 COVID-19 cases, including 89 deaths; 76.5% of cases occurred in wave 3 (*11*). 

**Figure 1:**
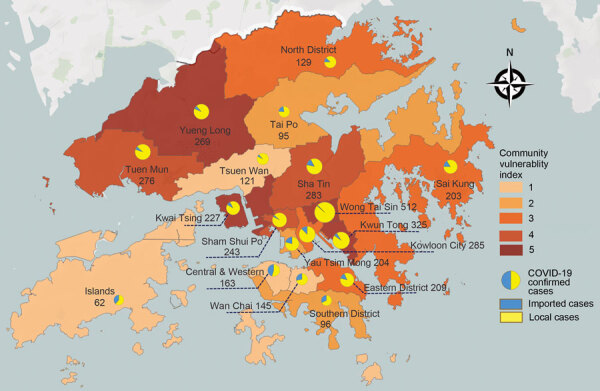
Distributions of community vulnerability index and total case counts of COVID-19 across administrative districts of Hong Kong as of August 31, 2020. COVID-19, coronavirus disease.

Following methods used by the Surgo Foundation (*6*), we first defined 5 domains that contributed to an overall CVI: socioeconomic status, household composition, housing condition, healthcare system, and epidemiologic factors. We included 22 indicators in the 5 domains for calculating domain CVI and overall CVI (Table 1). We first ranked each indicator by district, with a higher rank indicating greater vulnerability. Then, we calculated the percentile rank of each district over each indicator using the formula of percentile rank = (rank – 1)/(*n* – 1), where *n* refers to total geographic units (*n* = 18). A higher percentile rank indicates greater relative CVI of the district over the specific indicator. We then summed the percentile ranks over all indicators within each domain, reranked them to calculate domain CVIs, and summed the percentile ranks of all domains to calculate an overall CVI for each district. We assumed equal weights for indicators within domains and for the 5 domains within the overall CVI because of a lack of available evidence informing a more optimized weight scheme. Finally, we categorized all districts into very high (>80%), high (60%–80%), moderate (40%–60%), low (20%–40%), and very low (<20%) vulnerability on the basis of their domain and overall CVI. We calculated the Pearson correlations of indicator, domain, and overall CVI with COVID-19 case counts across districts and pandemic waves. We analyzed the differences in temporal trends of accumulated COVID-19 counts by districts of different vulnerability categories using Poisson regression models and plotted the results. We included 3,847 cases reported during January 23–August 31, 2020, for which a residence was locatable.

**Table 1:** Domains and domain indictors for calculating community vulnerability index in the context of the coronavirus disease pandemic, Hong Kong*

Domain	Descriptions of the indicators
Socioeconomic status†	
Poverty	Proportion of persons below poverty line‡
Unemployment	Proportion of persons >15 years of age who are unemployed
Income	Median income per capita
Educational level	Proportion persons >15 years of age having education level below high school
Household composition†	
Persons >65 years of age	Proportion of persons >65 years of age
Persons <14 years of age	Proportion of persons <14 years of age
Single-parent households	Proportion of single-parent households among all households
Elderly living alone	Proportion of elderly (>65 years of age) living alone
Housing conditions†	
Household density	Mean number of persons per household
Area of accommodation	Median floor area of accommodation per household
Healthcare system factors§	
Hospital beds	Proportion of hospital beds per 100,000 persons
Intensive care unit (ICU) beds	Proportion of ICU beds per 100,000 persons
Hospital labor	Proportion of hospital labor force (full-time staff employed by Hong Kong Hospital Authority) per 100,000 persons
Epidemiologic factors	
Population density¶	Estimated persons per square kilometre
Obesity#	Proportion of persons with BMI ≥25
Hypertension#	Proportion of persons with hypertension
Smoking#	Proportion of daily smokers
Persons employed in transportation sector†	Proportion of persons employed in transportation sector
Persons employed in accommodation and food catering sectors†	Proportion of persons employed in accommodation and food catering sectors
Working outside residency district†	Proportion of persons not working in the district of their residence
Entertainment venues**	Number of entertainment venues (e.g., bar, karaoke, wine, club house)
Non–Chinese ethnicities †	Proportion of persons with non–Chinese ethnicities

*All statistics were calculated at the residential district level of Hong Kong. †Data from the 2016 Hong Kong population by-census data (https://www.censtatd.gov.hk/hkstat/sub/so459.jsp). ‡Monthly household income is below 50% of the median monthly household income in Hong Kong before any government interventions. §Data from the 2018–2019 Hospital Authority Annual Report, Hong Kong (https://www3.ha.org.hk/data/HAStatistics/StatisticalReport/2018-2019). ¶Data from the Hong Kong Statistical Reports: Land area, mid-year population and population density by District Council district, 2019 edition (https://www.censtatd.gov.hk/hkstat/sub/sp150.jsp?productCode = D5320189). #Data from the 2015 report of a major Family Project Cohort Study comprising ≈8,000 households in Hong Kong (https://familycohort.sph.hku.hk/en/knowledge_exchange.html). **Data from OpenRice (https://www.openrice.com/en/hongkong), the most popular restaurant review app, which provides the largest database of food venues, bars and other entertainment venues in Hong Kong.

We plotted the spatial distribution of overall CVI and case counts (Figure 1) and domain CVI by districts (Appendix Figure 2). The 4 districts with very high vulnerability districts reported 1,333 COVID-19 cases, accounting for 34.6% (95% CI 33.1%–36.2%) of the total cases; the 4 districts with very low vulnerability reported 491 COVID-19 cases, 12.7% (95% CI 11.7%–13.9%) of the total. Of the 81 COVID-19–attributed deaths with recorded residence, 45.7% (95% CI 34.6%–57.1%) were reported in the 4 districts with very high vulnerability and 34.6% (95% CI 24.3%–46.0%) were reported in the 3 districts with high vulnerability. Only 2 COVID-19–attributed deaths were reported from the 7 very low or low-vulnerability districts.

**Figure 2:**
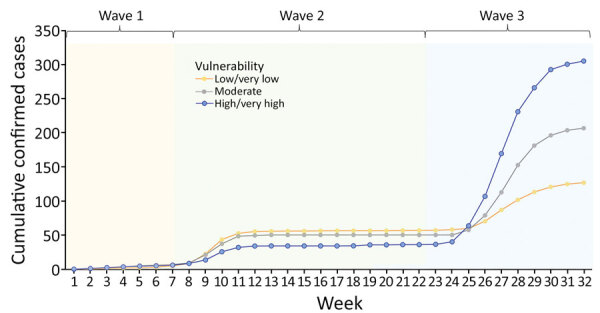
Cumulative coronavirus disease cases as of August 31, 2020, by week and districts of different vulnerability levels since the first case was reported in Hong Kong.

By pandemic wave, the correlation between overall CVI and case counts was not significant for wave 1, negative in wave 2, and positive in wave 3, and, consequently, positive overall (Table 2). In wave 2, the case counts correlated negatively with most indicator and domain CVI but correlated positively with distribution of entertainment venues and non-Chinese ethnicities. In wave 3, the case counts correlated positively with most indicators and all domain CVIs but correlated negatively with non–Chinese ethnicities. Overall, community profile variables that correlated positively with case counts over the 3 waves included poverty, income, educational level, single-parent households, area of accommodation, hospital beds, population density, obesity, and working outside area of residency.

**Table 2:** Pearson correlations of indicator, domain, and overall community vulnerability index with coronavirus disease confirmed cases across 3 pandemic waves, as of August 31, 2020, Hong Kong

Domains and indicators	Wave 1	Wave 2	Wave 3	Overall
Overall index	0.31	−0.49*	0.77‡	0.71†
Socioeconomic status	0.01	−0.59*	0.68†	0.58*
Poverty	0.26	−0.43	0.75‡	0.71†
Unemployment	−0.10	−0.64†	0.50*	0.38
Income	0.02	−0.65†	0.60^b^	0.48*
Educational level	−0.06	−0.70†	0.64†	0.51*
Household composition	0.14	−0.51*	0.57*	0.49*
Persons >65 years of age	0.59*	0.09	0.30	0.37
Persons <14 years of age	−0.36	−0.48*	−0.11	−0.25
Single-parent households	0.25	−0.44	0.73†	0.68†
Elderly living alone	−0.09	−0.28	0.45	0.41
Housing condition	0.16	−0.42	0.49*	0.43
Household density	0.07	−0.20	−0.01	−0.05
Area of accommodation	0.08	−0.32	0.65†	0.62†
Healthcare system factors	0.48*	−0.07	0.47	0.50*
Hospital beds	0.45	−0.33	0.59*	0.57*
ICU beds	0.41	0.16	0.39	0.47
Hospital labor	0.50*	−0.01	0.37	0.41
Epidemiologic factors	0.45	−0.37	0.68^†^	0.66^†^
Population density	0.45	0.17	0.51*	0.60^†^
Obesity	0.43	−0.32	0.53*	0.51*
Hypertension	0.54*	0.01	0.41	0.46
Smoking	−0.06	−0.51*	0.19	0.08
Employed in transportation sector	−0.22	−0.71†	0.26	0.10
Employed in accommodation and food catering sectors	−0.02	−0.51*	0.48*	0.39
Working outside residency district	−0.10	−0.56*	0.59*	0.49*
Entertainment venues	0.34	0.63†	0.04	0.21
Non–Chinese ethnicities	0.002	0.66†	−0.71†	−0.60†

*p<0.05 †p<0.01 ‡p<0.001

We plotted the temporal changes in cumulative cases by vulnerability categories (Figure 2). The Poisson regression model revealed an overall significant effect of vulnerability levels on cumulative cases (moderate vulnerability, β = 0.17, p<0.001; high/very high vulnerability, β = 0.31, p<0.001). By pandemic wave, districts of high/very high vulnerability reported fewer cases in the first 2 waves (β = −0.45, p<0.001) but significantly more cases in wave 3 (β = 0.68, p<0.001).

## Conclusion

Adding to existing literature (*2*–*5*), our study indicates that community vulnerability is dynamic, changing with the evolution of the pandemic. In waves 1 and 2, COVID-19 cases were mainly imported cases, including those infecting students and domestic helpers returning from overseas, as well as business travelers (*12*). These cases were found mainly within more socially privileged families and thereby an inverse association between socioeconomic status and case counts was seen. In Hong Kong, 55% of persons with non–Chinese ethnicities are domestic helpers for more socially privileged families and another 25% are executives or professionals (*13*) who have greater work-from-home flexibility (*12*). Subsequently, after tightening measures for inbound travelers and because there were more work-from-home arrangements in wave 3, the positive correlation between non–Chinese ethnicities and vulnerability to COVID-19 infection in waves 1 and 2 shifted to be negative. Entertainment venues constituted a primary exposure setting that spread COVID-19 in waves 1 and 2 (*14*) but ceased to be a major contributor to community vulnerability in wave 3 after these venues were closed. In wave 3, socioeconomic deprivation, poor housing, and dense household composition, as well as epidemiologic factors that facilitate viral transmission, became more key contributors to community vulnerability to COVID-19 infection. By April 2021, Hong Kong had experienced another pandemic wave (wave 4), characterized, again, by cases mainly in younger persons with higher socioeconomic status, linking to the largest local cluster (dancing/singing studio cluster) and the second largest cluster (fitness center outbreak) (*10*). Updated analyses found that socioeconomic deprivation and poor housing were no longer major contributors, whereas entertainment venues again became strong contributors to community vulnerability in wave 4 (Appendix Table). 

Overall, our study indicates that a COVID-19 CVI can be applied to district-level data within a city to help city-level policy makers in resource allocation planning, but these measures should be viewed as dynamic at different pandemic stages. For instance, infection control and prevention measures should be intensified, perhaps by more strict or substantial social distancing in community settings with entertainment venues where persons may remove their face masks to exercise, dance, or eat and drink when community incidence is lower to minimize pandemic resurgence, whereas more material resources can be allocated to support social distancing measures among more socially disadvantaged communities when widespread community outbreaks occur. Our analysis focused on the correlation of CVI with COVID-19 case counts rather than infection risk (i.e., incidence) or severity (e.g., fatalities) because of the relatively small number of cases and COVID-19 mortality in Hong Kong. However, because symptoms are generally mild in most cases, the magnitude of the pandemic impact is a key determinant for resource allocation.

Appendix Additional information on the community vulnerability index for COVID-19, Hong Kong.
