# Supplementary material for: Assessing Community Vulnerability over 3 Waves of COVID-19 Pandemic, Hong Kong, China
Source: Emerg Infect Dis. 2021 Jul;27(7):1935–9. doi: 10.3201/eid2707.204076 (PMC8237886; doi:10.3201/eid2707.204076)
Supplement: Appendix — Additional information on the community vulnerability index for COVID-19, Hong Kong. [file 20-4076-Techapp-s1.pdf]

Article DOI: https://doi.org/10.3201/eid2707.204076

Assessing Community Vulnerability over 3 Waves of COVID-19 Pandemic, Hong Kong, China

Appendix

Appendix Table. Pearson correlation of community vulnerability index (CVI) and indicators with COVID-19 confirmed cases over 4 waves of COVID-19 epidemic, Hong Kong (as of April 6, 2021)

Indicators	Wave 1*	Wave 2†	Original wave 3‡	Revised wave 3§	Wave 4¶	Overall
Community vulnerability index (CVI)	0.31	−0.49#	0.77††	0.69**	0.36	0.55#
Socioeconomic status	0.01	−0.59#	0.68**	0.69**	0.20	0.42
Poverty	0.26	−0.43	0.75c	0.77††	0.42	0.63**
Unemployment	−0.10	−0.64**	0.50#	0.51#	−0.10	0.12
Income	0.02	−0.65**	0.60**	0.62**	0.30	0.44
Educational level	−0.06	−0.70**	0.64**	0.66**	0.04	0.27
Household composition	0.14	−0.51#	0.57#	0.57#	0.19	0.36
Persons aged 65 and older	0.59#	0.09	0.30	0.28	0.02	0.18
Persons aged 14 or below	−0.36	−0.48#	−0.11	−0.11	−0.21	−0.25
Single-parent households	0.25	−0.44	0.73**	0.75††	0.44	0.63**
Elderly living alone	−0.09	−0.28	0.45	0.46	0.26	0.37
Housing condition	0.16	−0.42	0.49#	0.49#	0.04	0.23
Household density	0.07	−0.20	−0.01	−0.02	−0.33	−0.26
Area of accommodation	0.08	−0.32	0.65**	0.66**	0.47#	0.62**
Healthcare system factors	0.48#	−0.07	0.47	0.13	−0.05	0.02
Hospital beds	0.45	−0.33	0.59#	0.07	−0.17	−0.10
Intensive care unit (ICU) beds	0.41	0.16	0.39	0.57#	0.41	0.54#
Hospital manpower	0.50a	−0.01	0.37	−0.09	−0.14	−0.16
Epidemiologic factors	0.45	−0.37	0.68**	0.77††	0.73**	0.87††
Population density	0.45	0.17	0.51#	0.51#	0.82††	0.83††
Obesity	0.43	−0.32	0.53#	0.53#	−0.02	0.23
Hypertension	0.54#	0.01	0.41	0.40	0.64**	0.64**
Smoking	−0.06	−0.51#	0.19	0.21	−0.22	−0.10
Persons employed in transportation sector	−0.22	−0.71**	0.26	0.29	−0.36	−0.18
Persons employed in accommodation and food catering sectors	−0.02	−0.51#	0.48#	0.50#	0.56#	0.57#
Working outside of residency district	−0.10	−0.56#	0.59#	0.58#	−0.07	0.18
Entertainment venues	0.34	0.63**	0.04	0.04	0.67**	0.55#
Non–Chinese ethnicities	0.002	0.66**	−0.71**	−0.72**	−0.05	−0.32

*Wave 1, January 23–March 3, 2020.
†Wave 2, March 4–June 21, 2020.
‡Wave 3, June 22-August 31, 2020.
§Wave 3, revised, June 22–November 19, 2020.
¶Wave 4, November 20, 2020–April 6, 2021.
#p<0.05.
**p<0.01.
††p<0.001.

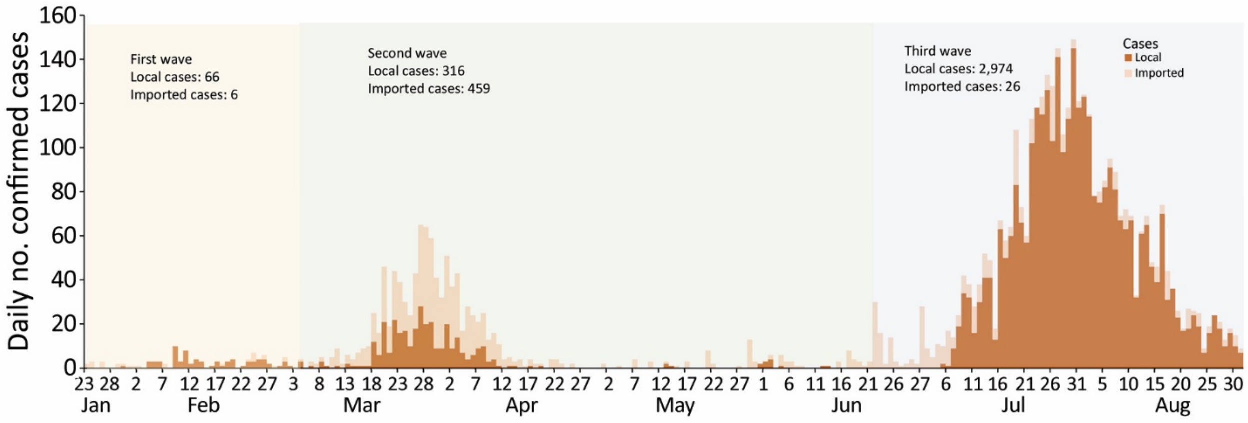

Appendix Figure 1. Daily number of COVID-19 cases as of August 31, 2020 over the 3 waves of COVID-19 pandemic in Hong Kong. Wave 1 (January 23−March 3, 2020) was characterized by a relatively stable and low number of cases, most of which were epidemiologically linked to cases imported from mainland China or the Diamond Princess cruise ship; wave 2 (March 4−June 21, 2020) was signified by predominantly imported cases from outside mainland China, including air crews and sailors, and precipitating several major clusters linked to social gatherings in entertainment venues; wave 3 (June 22−August 31, 2020) was characterized by a rapid increase in locally acquired cases and widespread community transmission following relaxation of most social distancing measures in June 2020.

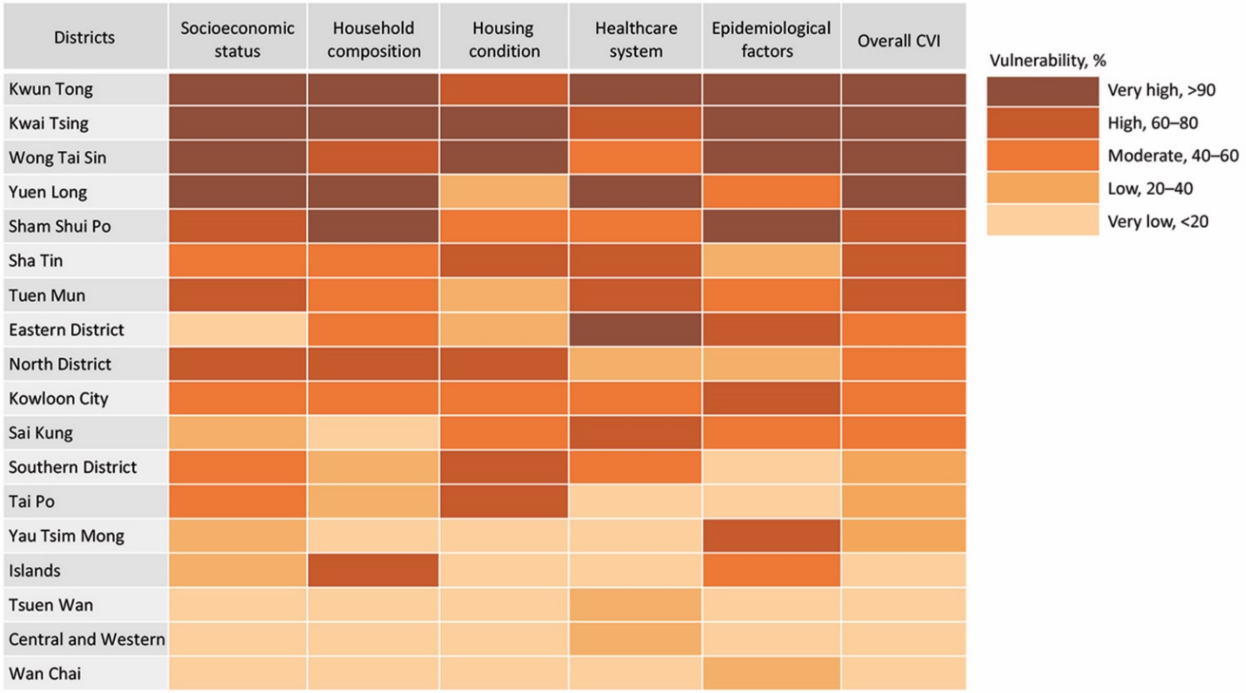

Appendix Figure 2. The vulnerability categories by domain and the overall CVI of the 18 districts of Hong Kong.
